# 机器人辅助与电视辅助胸腔镜肺段切除术治疗早期非小细胞肺癌短期效果比较

**DOI:** 10.3779/j.issn.1009-3419.2019.12.06

**Published:** 2019-12-20

**Authors:** 博恒 谢, 天一 隋, 毅 秦, 顺铖 苗, 文捷 矫

**Affiliations:** 266071 青岛，青岛大学附属医院胸外科 Department of Thoracic Surgery, Affiliated Hospital of Qingdao University, Qingdao 266071, China

**Keywords:** 机器人辅助胸腔镜手术, 电视辅助胸腔镜手术, 肺段切除术, 肺部结节, 短期效果, Robot-assisted thoracic surgery, Video-assisted thoracic surgery, Lung segmentectomy, Lung nodule, Short-term outcomes

## Abstract

**背景与目的:**

肺段切除术的开展越来越广泛，在现在的外科技术下，电视辅助胸腔镜手术（video-assisted thoracic surgery, VATS）是广泛采用的手术方式，同时机器人辅助胸腔镜手术（robot-assisted thoracic surgery, RATS）也提供了一种精准高效的手术方式，本研究旨在对比两种手术方式在肺段切除术中的短期效果。

**方法:**

回顾性分析2016年1月-2017年4月在青岛大学附属医院胸外科行VATS及RATS肺段切除术的患者临床资料，比较两组患者基线数据及手术时间、术中失血量、淋巴结切除数、术后胸腔引流管带管天数、术后住院天数、术后房颤及肺部感染发生率等。

**结果:**

共166例患者接受肺段切除术，两组均无中转开胸及围手术期死亡病例。其中VATS 85例，RATS 81例，术中淋巴结切除数在RATS组多于VATS组，差异具有统计学意义[（13.07±5.08）个*vs*（10.81±5.74）个，*P*=0.010）]，两组在手术时间、术中失血量、术后带管时间及住院时间无统计学差异（*P* > 0.05），术后的房颤、肺漏气（≥6 d）及术后肺炎发生率方面二组差异无统计学意义（*P* > 0.05）。

**结论:**

相比于VATS，RATS具有相似的安全性及可操作性，淋巴结切除数目较VATS明显为多。

## Abstract (en)

**Background and objective:**

Lung segmentectomy is increasingly used to resect lung nodules. Video-assisted thoracic surgery (VATS) is widely chosen to performing lung segmentectomy, while robotic assisted thoracoscopic (RATS) was also one useful and practical method. There article was intended to compared the short-time outcomes of RATS and VATS in lung segmentectomy.

**Methods:**

The patients with lung nodules underwent segmentectomy by either RATS or VATS from January 2016 to April 2017 were studied. Baseline characteristics and short-time outcomes (dissected lymph nodes, postoperative duration of drainage, postoperative hospital stay, incidence of pro-longed air leak, atrial fibrillation and pneumonia) were compared.

**Results:**

166 patients were included in this study: 81 patients underwent RATS segmentectomy while 85 underwent VATS segmentectomy. The number of lymph nodes dissected in RATS group was more than in VATS group [(13.07±5.08) *vs* (10.81±5.74), *P*=0.010]. The incidence of some postoperative complications such as pro-longed air leak, atrial fibrillation was not significant different between the two approaches.

**Conclusion:**

Compared with VATS, RATS has similar safety and operability, and the number of lymphadenectomy is significantly more than that of VATS.

肺癌目前是中国发病率及致死人数排在第一位的恶性肿瘤^[1]^，目前，手术仍然是有早期肺癌治疗的主要方法。肺叶切除术+淋巴结清扫术作为可手术肺癌患者治疗的金标准，已经开展了多年^[2]^，现在随着低剂量螺旋计算机断层扫描（computed tomography, CT）普查的开展及普查人群的扩大，肺癌的整体预后正在逐渐改善，早期肺癌患者占比逐渐增多^[3]^。对早期非小细胞肺癌，肺段切除术一方面能够提供与肺叶切除术相近的预后效果，并且能够保留更多的健康肺组织^[4]^，有更好的肺功能和更佳的生活质量^[5, 6]^，因此肺段切除术在早期肺癌中的应用逐渐增多。随着外科技术与器械的快速进步，机器人辅助胸腔镜手术（robot-assisted thoracic surgery, RATS）正在胸外科领域得到更多应用^[7]^。作为微创胸外科手术的一种，RATS与电视辅助胸腔镜手术（video-assisted thoracic surgery, VATS）是都是进行肺手术安全有效的手术方式，二者在淋巴结清扫站数、术后并发症等方面均优于开放手术^[8]^。VATS及RATS进行肺段切除术的研究仍然比较有限，RATS和VATS二者在肺段切除术中的安全性、有效性方面的对比尚不充分，目前RATS与VATS在肺手术中作用的对比研究常含有肺叶切除与肺段切除两种手术方式，不能完全体现肺段切除术的特点，需要进一步研究。

## 1 资料与方法

### 1.1 一般资料

选取2016年1月-2017年8月间在青岛大学附属医院胸外科以RATS及VATS两种手术方法实施肺段切除术的患者，其中接受RATS组81例，VATS组85例。肺段切除术选择标准：①心肺功能无法耐受肺叶切除术或有再次手术可能，选择肺段切除。②CT提示为非侵袭性病变（指位于肺实质外侧2 cm，病变直径 < 2 cm）并且具有以下特征：病理证实为原位腺癌（adenocarcinoma *in situ*, AIS）；CT随访1年以上高度怀疑为恶性肿瘤，磨玻璃样成分≥50%，影像学检查直径倍增时间≥400 d。患者在年龄、性别等基线信息方面的差异无统计学意义（*P* > 0.05）。对于术前影像学检查结节直径≤2 cm且为纯磨玻璃结节者术前检查包括心脏超声及肺功能检查以评功心肺功能和颅脑磁共振成像（magnetic resonance imaging, MRI）。上腹部CT、甲状腺及颈部彩超及骨扫描或正电子发射型计算机断层显像（positron emission computed tomography, PET）/CT用于结节中出现实性成分者以排除潜在转移可能性。患者一般临床资料见表 1。

**1:** 患者一般临床资料比较 Characteristics of patients

Index		Robotic-assisted thoracic surgery	Video-assisted thoracic surgery	*t*	*P*
*n*		81	85		
Gender	Male	38	36		
	Female	43	49		
Age, yr, median (range)		59.01 (33-78)	59.14 (28-75)	0.866	0.300
Benign		4	7		
Metastasis tumor		2	2		
Non-small cell lung cancer	Ia1	40	43		
	Ia2	31	26		
	Ia3	3	5		
	Ib	1	2		

### 1.2 手术方法

所有手术方式均由同一组医师完成，RATS系统为达芬奇机器人系统（Da Vinci Robotic System）（Intuitive Surgical, Inc, Mountain View, California, USA）。胸腔镜选用德国KARL STORZ高清胸腔镜系统。二种手术均采用双腔气管插管，健侧90°侧卧于手术台，折刀体位。根据肺裂发育情况选择处理顺序，采用标准肺叶切除术方式处理肺门组织，对直径2 mm以下血管采用超声刀（VATS）或电凝（RATS）处理，对于2 mm及以上血管，VATS术中以上常规进行结扎后切断或直线切割闭合器切断，RATS术中应用Hem-O-Lock夹闭后切断。肺段间平面选择充气-萎陷法确定，段间组织选择直线切割闭合器及超声刀切断。术后剖检标本，保证结节边缘据肺段切缘≥20 mm或超过结节直径。对于为AIS或微浸润腺癌进行采样，其他类型进行系统淋巴结清扫，并常规送检第12组淋巴结冰冻病理，第12组淋巴结转移阳性者改行肺叶切除术+系统性淋巴结清扫术。手术部位肺段具体情况见表 3。

**3:** 两组患者术后相关资料比较 The postoperative clinical data between the two groups

Variations	Robot-assisted thoracic surgery (*n*=81)	Video-assisted thoracic surgery (*n*=85)	*t*	*P*
Diameter	1.35±0.76	1.54±0.84	-1.550	0.094
Blood lost (mL)	53.46±30.01	46.00±47.44	1.200	0.230
Operating time (min)	126.46±25.02	122.2±20.32	1.334	0.184
Lymph nodes harvested	13.07±5.08	10.81±5.74	2.060	0.010
Duration of chest driange (d)	2.79±1.7	2.66±1.47	0.583	0.561
Pro-longed air leak	3.7% (3/81)	3.5% (3/85)	1.000	0.952
Atrial fibrillation	2.47% (2/81)	3.5% (3/85)	1.000	1.000
Post-operative hospital stay (d)	5.05±1.91	5.17±1.60	-0.480	0.632

### 1.3 统计学方法

所有数据应用SPSS 22.0软件进行分析，χ^2^检验或*Fisher*精确概率法用于比较两组各并发症发生率的差异，*t*检验用于比较连续变量的均数差异。*P* < 0.05认为具有统计学差异。

## 2 结果

两组患者均顺利完成手术，无中转开胸病例，无围手术期死亡病例。手术结果见表 2。连续变量以均数±标准差的方式表示，分类变量用百分比表示。在淋巴结切除数目方面，RATS组较VATS组为多，差异具有统计学意义[（13.07±5.08）个*vs*（10.81±5.74）个，*P*=0.010]。而在手术时间[（126.46±25.02）min *vs* （122.2±20.32）min, *P*=0.184]、术中失血量方面[（53.46±30.01) mL *vs*（46.00±47.44）mL, *P*=0.230]，RATS组多于VATS组，但差异不具有统计学意义（*P* > 0.05）。术后并发症方面，术后房颤发生率（2.47% *vs* 3.5%）VATS组稍高，而肺漏气发生率（2.47% *vs* 3.5%）RATS组稍高，但差异均无统计学意义（*P* > 0.05），见表 3。

**2:** 肺段手术资料 Features of segmentectomies

Status of segments	Robotic-assisted thoracic surgery	Video-assisted thoracic surgery
S1+2+3	11	13
S4+5	9	10
S6	11	11
S7+8+9+10	1	2
S1	6	7
S1+2	12	13
S2	11	12
S3	9	7
S1+3	7	8
S3+4+5	2	2
S1a+S2	1	0
S8	1	0

## 3 讨论

RATS与VATS在研究中最明显的不同在于淋巴结切除数方面，RATS在淋巴结切除数方面较VATS为多，两种手术方式切除的淋巴结数目对比有明显的统计学差异（*P*=0.010）。淋巴结切除术在肺癌手术治疗中具有重要的作用，良好的淋巴结切除对于非小细胞肺癌的准确临床分期及指导后续治疗非常重要^[9]^。Krantz等^[10]^研究表表明，肺段切除术治疗非小细胞肺癌，切除的淋巴结数目超过14枚者，其预后较少于14枚的患者更好。因此，切除更多的淋巴结，对于预后可能有着积极影响。Toker等^[11]^报道了15例RATS肺段切除术，其纵隔淋巴结获取数目及肺门与叶间淋巴结数目分别为14.3（2-21）个和8.1（2-19）个，但其手术例数较少。在既往的文献中，RATS对于淋巴结处理的更大优势在于淋巴结切除的站数等。相比于开放手术，RATS及VATS在淋巴结切除方面展示了比较明显的优势，在淋巴结切除的数目、站数方面均优于开放手术^[8]^。在Yang等^[12]^进行的RATS对比纯单孔VATS肺切除术的研究中，发现RATS在淋巴结站数的切除数方面较纯单孔VATS淋巴结站数有优势，但是切除的淋巴结数目方面，两组之间的差异并无统计学意义（*P* > 0.05），其结论与本研究结果略有不同。在与VATS肺叶切除术的比较中，RATS可以达到更高的淋巴结清除率，对于淋巴结状态的评估有利^[13]^。上述研究均提示RATS在切除淋巴结方面的潜力。我们认为出现此结果的原因主要在于相比于肺叶切除术，接受肺段切除术的患者一般属于较早期患者。因此，有时术者会倾向于选择进行淋巴结采样而非淋巴结清扫术，对于一些清扫部位困难的淋巴结，比如紧贴动肺段动脉的淋巴结，术者可能出于安全的目的，放弃切除或者选择部分采样。相比于VATS，RATS拥有VATS难以匹敌的全向机械关节优良且能够放大的3D视野，并且能够完全过滤术者手臂的颤动，能够极大地改善术者的手术体验，并且赋予术者更充分的自信。Mungo等^[14]^报道相比VATS，RATS能够有效减少手术中难以控制的出血的发生。这使得对于一些位置，比如紧贴于动静脉表面的淋巴结的切除，术者有能力并且愿意去冒险切除。虽然，RATS无法提供VATS中一样的手指触觉，并且缺乏真正的触觉力反馈。但是，这些不足，相比于RATS系统提供的优势并不会影响肺癌手术中淋巴结的清扫。

RATS在所用的手术时间上（切皮-缝合时间）略超过VATS，二者之间并无明显的统计学差异，我们认为这是因RATS操作台安装时间较长所致。在既往文献中，不同作者报道的VATS肺段切除术和RATS肺段切除术在手术时间上的比较结果也有不同。Rinieri等^[15]^比较了17例RATS肺段切除术与34例VATS肺段切除术，二者平均手术时间分别为150（120-180）min和150（120-180）min，差异无统计学意义（*P*=0.507）。而由Demir^[16]^研究的34例RATS肺段切除术与65例VATS肺段切除术，二者平均手术时间为（76±23）min和（65±22）min，其差异具有明显统计学意义（*P*=0.018）。之所以出现这种不同研究结论间的差异，我们认为一方面是各研究之间选择的病例数目不同，以及部分研究属于初步开展时进行的研究，易受到学习曲线的影响。另一方面，研究中选择的肺段部位不同，对于结果可能会有一定影响。最近Handa等^[17]^对比研究了不同类型肺段切除术手术时间的关系，将肺段切除术分为复杂与简单两类，两种不同类型肺段切除术之间相比，复杂肺段手术时间明显超过简单肺段的手术时间，二者对比有差异（180 min *vs* 143.5 min, *P* < 0.001）。在研究中不同类型肺段所占比例不同，对最终手术时间的对比可能产生不同的影响。

在本次研究中，两种手术方式共纳入了166例肺段切除术，在我们的研究中，二者在术后胸管带管时间、术后住院时间方面也没有发现明显的统计学差异。这与代锋等^[20]^的研究结果相同，认为RATS不会延长术后带管时间，不会增加术后胸腔积液引流量。RATS与VATS两种手术方式术后并发症主要有房颤、肺部感染、漏气。二者在具体并发症方面的比较，均未发现明显的统计学差异，这与既往文献^[15, 16]^所报道的相似，RATS和VATS肺段切除术在术后并发症的发生率方面并无明显差异。

即使现在肺段切除术研究已经比较充分，整体也已经比较安全，但是不管是RATS还是VATS，在肺段切除过程中，都存在着各种可能导致切除范围扩大转行肺叶切除术的可能。肺段切除术过程中，RATS术中无法触摸结节，没有真实触觉，使较小的肺表面结节的定位略困难，且仅能获得一定的视觉反馈，这是RATS的缺点，可能导致在进行手术时对于结节与肺段间隙的关系不易把握，对术者的经验和技术提出了比较高的要求。Cerfolio等^[21]^研究了连续100例的术前计划行RATS肺段切除术的患者，发现其中7例转行了肺叶切除术，没有中转开放手术的病例。7例患者原因分别为：4例患者切缘不充分，1例患者术后在一个肺段标本中无法定位结节，2例患者无法通过肺段切除术完成手术（结节位置不适）。RATS的优势也表现在肺手术中损伤血管引起大出血的几率低于VATS^[22]^，这也是术者手术信心的来源。RATS通过机械臂的灵活运动，尤其是术者一个人即可操控机械臂，实现对肺叶的翻动和肺门结构的暴露，可以更精确地施行手术。术者坐位操作能够节省体力，更适合较长时间进行手术，助手可以通过辅助切口或其中一个操作孔操作吸引器或直线切割闭合设备。不同于VATS需要专门的扶镜助手，RATS术中术者可随心所欲控制镜头，能够减少助手因素对手术造成的影响，保证连续手术的治疗。

RATS相比于VATS，具有相似的安全性及可操作性，淋巴结切除数目较VATS明显为多。二者在手术时间、术中出血量、术后住院时间结果相似并且未增加术后肺炎、房颤、术后肺漏气等并发症的发生率。但是此项研究并未涉及患者的长期生存和复发情况，因此RATS肺段切除术在淋巴结切除数目方面的优势是否能够带来长期生存方面的获益尚不明确。并且RATS系统价格昂贵，对于运营的医疗机构以及接受该系统治疗的患者都是如此，虽然目前RATS逐渐得到更广泛的推广应用^[20]^，但仍有众多问题尚未得到解决，尤其是在预后的影响方面还需要更进一步的研究。因此，更多前瞻性地临床研究有待开展，以继续揭示机器人系统在非小细胞肺癌手术治疗中的作用。
